# Composition, thickness, and homogeneity of the coating of core–shell nanoparticles—possibilities, limits, and challenges of X-ray photoelectron spectroscopy

**DOI:** 10.1007/s00216-022-04057-9

**Published:** 2022-04-26

**Authors:** Jörg Radnik, Xenia Knigge, Elina Andresen, Ute Resch-Genger, David J. H. Cant, Alex G. Shard, Charles A. Clifford

**Affiliations:** 1 Bundesanstalt für Materialforschung Und -Prüfung (BAM), Division 6.1 “Surface Analysis and Interfacial Chemistry”, Unter den Eichen 44-46, 12203 Berlin, Germany; 2 Bundesanstalt für Materialforschung und -Prüfung (BAM), Division 1.2 “Biophotonics”, Richard-Willstätter-Str. 11, 12489 Berlin, Germany; 3 National Physical Laboratory, Surface Technology Group, Hampton Road, Teddington, TW11 0LW UK

**Keywords:** X-ray spectroscopy (XPS/XRF/EDX), Nanoparticles/nanotechnology, Spectroscopy/instrumentation, Spectroscopy/theory

## Abstract

Core–shell nanoparticles have attracted much attention in recent years due to their unique properties and their increasing importance in many technological and consumer products. However, the chemistry of nanoparticles is still rarely investigated in comparison to their size and morphology. In this review, the possibilities, limits, and challenges of X-ray photoelectron spectroscopy (XPS) for obtaining more insights into the composition, thickness, and homogeneity of nanoparticle coatings are discussed with four examples: CdSe/CdS quantum dots with a thick coating and a small core; NaYF_4_-based upconverting nanoparticles with a large Yb-doped core and a thin Er-doped coating; and two types of polymer nanoparticles with a poly(tetrafluoroethylene) core with either a poly(methyl methacrylate) or polystyrene coating. Different approaches for calculating the thickness of the coating are presented, like a simple numerical modelling or a more complex simulation of the photoelectron peaks. Additionally, modelling of the XPS background for the investigation of coating is discussed. Furthermore, the new possibilities to measure with varying excitation energies or with hard-energy X-ray sources (hard-energy X-ray photoelectron spectroscopy) are described. A discussion about the sources of uncertainty for the determination of the thickness of the coating completes this review.

**Supplementary Information:**

The online version contains supplementary material available at 10.1007/s00216-022-04057-9.

**Figure:**
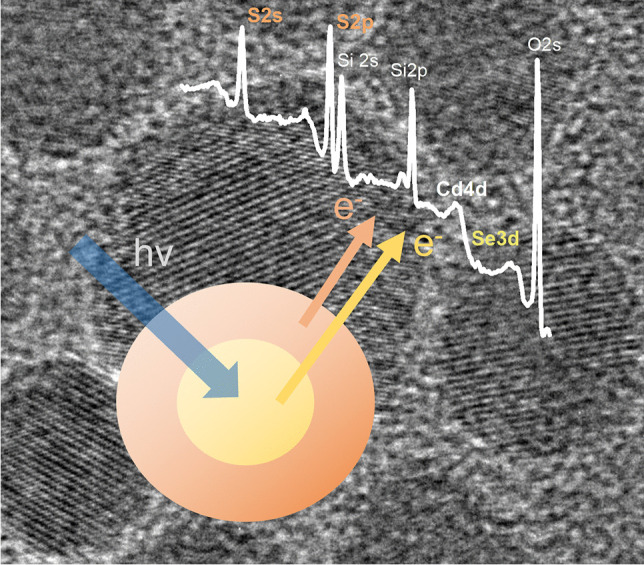
Graphical abstract

## Introduction

The encapsulation of nanoparticles by a shell or a coating is often crucial for their stability and for all properties where the composition, thickness, and homogeneity of the shell determine the interaction of the nanoparticles with their environment. Examples of the application of such core–shell nanoparticles are found in biomedicine, e.g. for drug delivery and specific targeting, for bioimaging, as transplant material, in catalysis, or in electronics [1].

Whereas methods for size and shape determination of nanoparticles such as (high-resolution) transmission electron microscopy ((HR-)TEM), scattering techniques like small- and wide-angle X-ray scattering (SAXS and WAXS), and dynamic light scattering (DLS) and small-angle neutron scattering are well established [2], methods for determining the chemical nature of nanoparticles are still rare. One method which can be used is X-ray photoelectron spectroscopy (XPS) which can provide information on the chemical composition and the amount of chemical groups or certain chemical species in the near-surface region of the particles, even for light elements [3].

Due to its high surface sensitivity, XPS is ideally suited for the investigation of the coating of core–shell nanoparticles often with a thickness of few nanometres. This thickness corresponds with the typical information depth of lab-based instruments using Al Kα X-rays (hν = 1486.6 eV) which is governed by the attenuation of the photoelectrons ejected from the electron shell under exposure to an X-ray beam. Measuring the intensity of these photoelectrons provides quantitative information about the surface composition and binding state information, i.e. the amount of chemical groups or species at the surface of the sample. Attenuation is determined by the sample material and the kinetic energy of the ejected electrons. Deeper regions of the particles can be reached with the use of higher energy X-ray sources which have become available in the last few years in laboratories or using synchrotron radiation sources.

Although XPS is an inherently nanoscale technique in terms of the depth of analysis, it is an ensemble technique for nanoparticles due to an analysis area ranging from several square micrometres to approximately 0.5 mm^2^ depending on either the spot size of the micro-focussed X-ray beam or the apertures and lenses limiting the analysis area. Thus, the results are an average of the nanoparticles within the analysis area, and homogeneity of the analysed nanoparticles is often assumed when interpreting the data.

Confirmation of this homogeneity can be investigated with electron microscopy, which can give information about the size, morphology, and structures of the nanoparticles. Coupled with energy-dispersive X-ray spectroscopy (EDS), elements typically heavier than Na can be detected. In contrast, XPS provides information about compounds of light elements which are typical for organic coatings, such as ligands or biomolecules. Additionally, the valence states of the components can be investigated which permits chemical structure determination. Therefore, information can be obtained with XPS that is not available with electron microscopy or other methods like SAXS [4–7]. Thus, XPS is complementary with methods for the investigation of the size, shape, or morphology of the particles. Correlating XPS results with the results of these provides comprehensive insights into the core–shell structure of nanoparticles. Such correlation of HR-TEM with XPS investigations was used for elucidating the core–shell architecture of nanometre-sized quantum dots (QDs). QDs with their size-tuneable optical properties are widely used in different industries including biosensing, display technology, solid-state lighting, and solar energy conversion [8, 9]. In these spherical or elongated nanostructures, a thick inorganic passivation shell shields the core, which is crucial for high photoluminescence quantum yields close to unity, providing excellent photostability [10–12]. The surface passivation shell is commonly covered by coordinatively bound organic ligands. The chemical nature of the stabilizing ligands is also relevant because they determine colloidal stability, processability, e.g. the incorporation into matrices for device fabrication, and functionalization [13, 14]. Due to the importance of the particle architecture, reliable methods are needed to assess relevant features such as the size of the particle and the core, and the thickness and chemical composition of the inorganic passivation shell [1, 15]. In addition, information on the occurrence and amount of core–shell intermixing are increasingly desired to fine-tune synthetic procedures and optimize material performance [16].

**Fig. 1:**
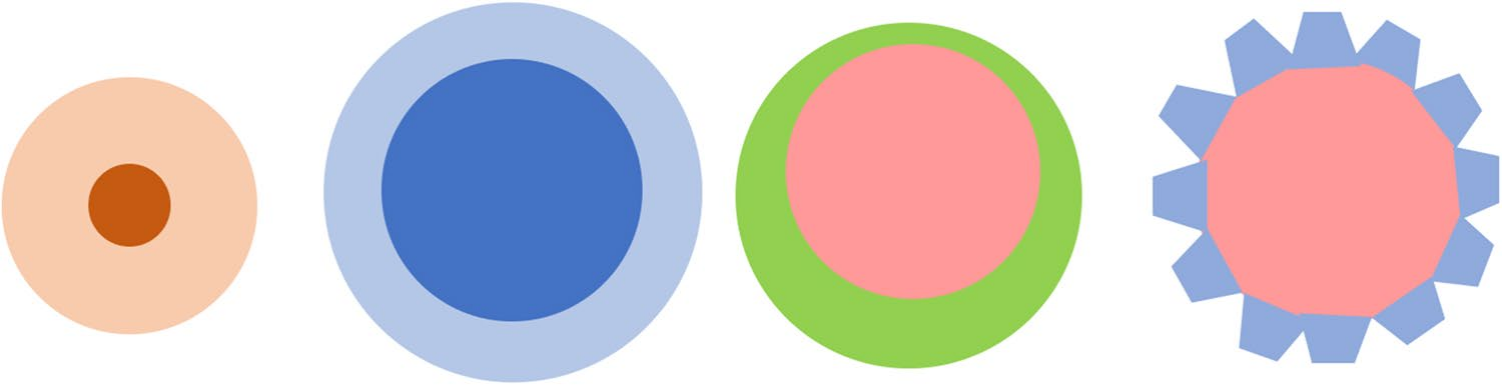
Scheme of the nanoparticles presented in this review. The size, coating, and core are described in Table 1 in the same order as in the figure

In this paper, we detail four examples of core–shell nanoparticles analysed with XPS and hard-energy X-ray photoelectron spectroscopy (HAXPES) as summarised in Table 1 and Fig. 1. Firstly, QDs with CdSe core and a CdS shell are presented as an example of a small nanoparticle of ca. 10 nm with a thick inorganic coating. Furthermore, the core–shell intermixing is investigated here with XPS.

**Table 1:** Overview of the investigated nanoparticles

Size	Small (10 nm)	Medium (40 nm)	Medium–large (50–100 + nm)	Medium–large (50–100 + nm)
Core	Inorganic (CdSe)	Inorganic (NaYF:Yb)	Organic (PMMA)	Organic (PS)
Coating	Inorganic (CdS)	Inorganic (NaYF_4_:Er)	Organic (PTFE)	Organic (PTFE)
Organic ligand	Oleic acid/oleylamine	Oleic acid

Secondly, NaYF_4_-based upconverting nanoparticles doped with Yb^3+^ as sensitizer ions and Er^3+^ as activator ions have been investigated. In these upconverting nanoparticles (UCNPs), the sensitizer ions absorb two or more near-infrared photons and transfer the energy to the activator ions which emit multiple element-specific bands in the ultraviolet, visible, and near-infrared [17–19]. Crucial for the underlying energy transfer processes and the upconversion efficiency are the internal spatial distribution and concentration of activator and sensitizer ions. This is because an appropriate distribution can prevent surface quenching, i.e. the quenching of the lanthanide emission at the surface by defect states and high-energy vibrational modes from -OH, -NH, or -CH groups from the organic ligand shell and/or solvent molecules and/or reduce concentration quenching as well as controlling the energy migration pathways within the UCNPs. These nanoparticles were four times larger than the CdSe—CdS QDs with a comparatively thin coating. Here, the combination of XPS and HAXPES was used to analyse the internal spatial distribution of the absorbing sensitizer ion and the optically active activator ion in UCNPs. Traditional XPS is sensitive to the outermost ~ 5 nm of the particles. For particles larger than 10 nm like the upconverting nanoparticles, the coating features are dominant. Whereas with HAXPES, both the coating and the core were measured, so it is possible to distinguish between these different regions.

Polymer nanoparticles attract increasing attention due to their use for life sciences, bioimaging, therapeutics, and sensing applications [20, 21] as well as the potential impact of nanoplastics on the environment [22]. To understand the interaction between particles and their environment, a detailed knowledge of the chemical composition and thickness of the coating is crucial. Electron microscopy can only provide limited knowledge about these properties because it is not chemically sensitive to organic compounds, and the very slight contrast difference between core and coating with similar light elements makes it difficult to distinguish these two particle regions. Usually, the thickness measurement of the coating is performed in two separate steps: in the first step, the diameter of particles consisting only of the core is measured. In a separate step, the diameter of the whole core–shell nanoparticles is determined. Half the difference between these two diameters is specified as the nominal thickness of the coating. Inhomogeneities of the coating or intermixing between core and coating are not considered in this approach. For size determination, SAXS or electron microscopies like SEM or TEM can be used.

In this review, we present two types of polymeric particles with a non-centric poly(tetrafluoroethylene) (PTFE) core. One of these has a coating of poly(methyl methacrylate) (PMMA) with varying nominal thicknesses and another one with an incomplete polystyrene (PS) coating, also with varying nominal thicknesses. These examples illustrate the challenges of measuring non-ideal core–shell nanoparticles (Fig. 1 and Table 1).

## Theoretical background

For flat uniform samples, the measurements of the composition and the thickness of overlayers with XPS are well established. A formula for the calculation of the thickness of oxide overlayers was developed in the 1970s [23] and the methods are standardized [24]. For samples which are not flat, these methods will be in error because the sample geometry must be considered, especially for particles with sizes in the same length scale as the inelastic mean free path of electrons. This means that for nanoparticles, not only the shell on the topside of the particle must be considered, but also on the sides and underside of the particles. Potentially even particles beneath the outermost layer of the nanoparticles can contribute to the measured signal.

To account for the effect of sample geometry on the determination of the thickness and chemical nature of nanoparticle coatings, several methods have been reported with varying degrees of accuracy. Ultimately, these methods are derived from modelling the expected photoelectron intensities detected from a given sample. These methods can be broadly generalised into (a) simple numerical modelling, in which a number of assumptions are applied, allowing estimation of intensities to be performed using simple scripts or spreadsheets; (b) descriptive formulae, whereby these calculations have been simplified further into a set of empirically derived formulae for direct calculation of overlayer thicknesses; and (c) comprehensive simulation, involving the use of expert-designed software [25]. In order to model non-ideal nanoparticle structures such as those outlined in Fig. 1, either (a) simple numerical modelling or (c) simulation packages must be used, as descriptive formulae typically require a known or assumed concentric structure. These two are detailed below along with information that can be determined from inelastic background modelling.

### Simple numerical modelling

For the majority of nanoparticulate systems, the use of simple numerical modelling is more than adequate to provide an estimate of features such as coating thickness and composition. When performing these calculations, a number of assumptions are typically used; first and foremost, the ‘straight-line approximation’ is applied [26], i.e. it is assumed that all electrons that were emitted and eventually detected have travelled in a straight line through the sample from ejection to the detector, without elastic scattering. This assumption provides an acceptable approximation for the majority of materials but can result in a reduced accuracy for nanoparticle systems bearing a highly scattering coating, typically those materials composed of heavier elements [27]. For simplicity, it is usually assumed that the XPS intensities measured from a nanoparticle sample will be proportional to the intensities originating from a single nanoparticle. This assumption is valid for samples in which the nanoparticles are randomly arranged [28].

Given these assumptions, the expected intensities of core levels from a nanoparticle system can be modelled by calculating the intensity from individual points on a nanoparticle as if they were a standard flat overlayer/substrate using Eqs. (1) and (2), respectively, and summing the resulting intensities over the projected area of the nanoparticle [29–34].

1 $${I}_{x}={I}_{x,\mathrm{X}}\left[1-{\mathrm{e}}^{\left(\frac{-\mathrm{a}}{{\mathrm{L}}_{\mathrm{x},\mathrm{X}}}\right)}\left(1-{\mathrm{e}}^{\left(\frac{-\mathrm{b}}{{\mathrm{L}}_{\mathrm{x},\mathrm{Y}}}\right)}\left(1-{\mathrm{e}}^{\left(\frac{-\mathrm{c}}{{\mathrm{L}}_{\mathrm{x},\mathrm{X}}}\right)}\right)\right)\right]$$

2 $${I}_{y}={I}_{y, Y}{e}^{\left(\frac{-a}{{L}_{y,X}}\right)}\left(1-{e}^{\left(\frac{-b}{{L}_{y,Y}}\right)}\right)$$

where

- *X* and *Y* subscripts refer to the materials of the coating and the core, respectively
- *x* and *y* subscripts refer to the specific photoelectron peaks from materials X and Y
- ${I}_{j}$ is the intensity of electrons arising from a core level, *j*
- ${I}_{j,J}$ is the intensity of electrons from peak *j* arising from a pure reference material *J*
- $a$ is the vertical thickness of the coating material on top of the core at a given position
- $b$ is the vertical thickness of the core material at a given position
- $c$ is the vertical thickness of the coating material underneath the core at a given position
- ${L}_{j,J}$ is the effective attenuation length of electrons from core level $j$ travelling through material $J$

**Fig. 2:**
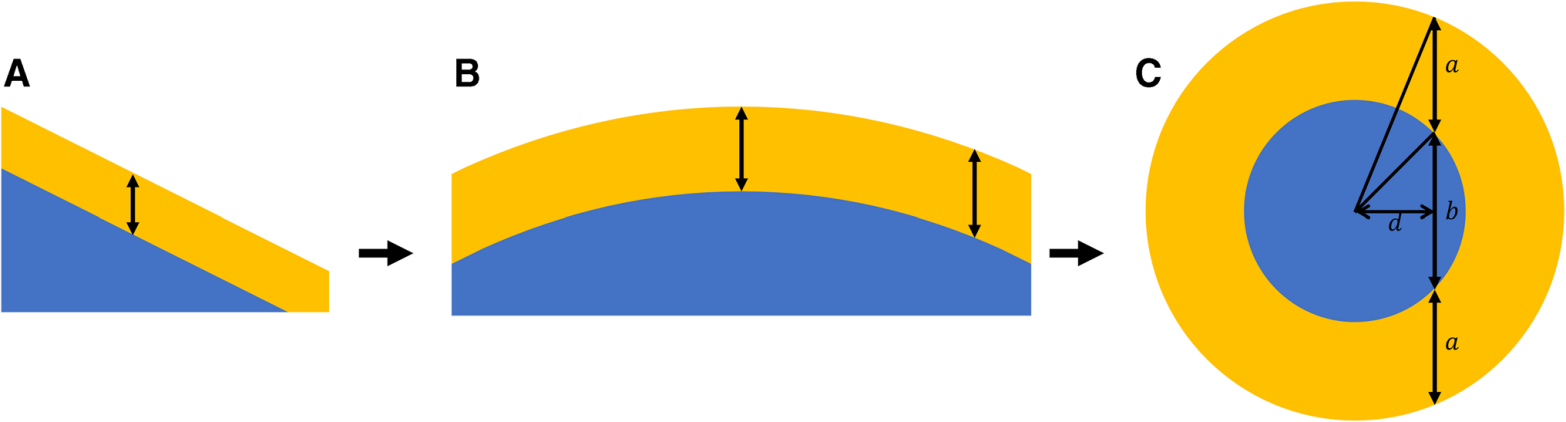
An example of a flat sample at an angle **(A)**. For macroscopic topography, the thickness at each point on the surface must be determined—for generic shapes, there exist topofactors which simplify this process **(B)**. For nanoparticles, the intensity from each point on the surface (i.e. all values of *d*) must be considered, including contributions from the non-negligible coating at the sides and beneath the core **(C)**

Figure 2 depicts the differences between three situations of coatings on (A) a flat surface, (B) a macroscopically curved surface (e.g. surface-modified microparticle), and (C) a spherical nanoparticle. For a flat surface, a simple calculation of the effective vertical thickness can be done based on the sample angle. For a topographic surface with a uniform coating, e.g. macroscopic spheres, this must be modelled for each point in the surface—for generic shapes such as spheres and cylinders, this can be approximated with a ‘topofactor’; in these cases, the diameter of a macroscopic particle is not important and the topofactor depends only upon the specific particle shape. For nanoparticle samples, it is also necessary to consider contributions from coating at the sides and beneath the core—therefore, each system must be modelled completely, with expected intensities summed across every point on the surface of the particle to generative expected intensity ratios. This process has been described in more detail in an ISO technical report on nanoparticle coating analysis [29, 30].

This type of modelling for core–shell nanoparticles has been used for a number of purposes, including direct analysis of nanoparticle systems [35], development of descriptive formulae [5, 36], and it can be adapted for use with ‘non-standard’ morphologies [37]. In the latter case, it is possible to use such methods to compare measured XPS intensities with those expected from a specific morphology (e.g. a non-central core) by appropriate consideration of the geometry—there may however be several morphologies that may produce identical XPS peak intensity ratios, so it is crucial that such models are supported by other measurements.

### Simulation

In cases in which greater accuracy may be required, there are simulation packages available for more sophisticated modelling of XPS intensities and spectra. Such packages may be required in cases where elastic scattering is significant and the straight-line approximation loses validity, or where more complex nanoparticle structures are assessed, including systems for which the single-particle approximation may not be valid, such as those with large-scale order. Currently, the most well-known simulation package is the NIST Database for the simulation of electron spectra for surface analysis (SESSA) [25]. SESSA itself contains databases of important parameters for performing simulations of XPS spectra, including inelastic mean free paths, photoionisation cross sections, elastic scattering cross sections, and asymmetry parameters. The capability of software such as SESSA for determining structural information of nanoparticle systems from XPS data has been repeatedly demonstrated in the literature, both for ideal concentric cases and for heterogeneous or non-uniform samples [7, 38–40]. In general, it has been found that for simple cases, the use of simple numerical models, descriptive formulae, and simulation using SESSA provides agreement within the uncertainty of the electron attenuation length, with discrepancies primarily arising for cases with significant elastic scattering, where SESSA provides more accurate data [25].

### Inelastic background modelling

While most XPS analyses focus on the measurement of peak intensity ratios to determine the relative concentrations of the components in the near-surface region, there has also been a significant amount of work on the analysis of the inelastic background signal. In particular, the background shape helps to conclude whether a given material is present as a surface coating or shell, or as a substrate or core material. In simple terms, overlayer materials produce a minimal increase in the background signal at kinetic energies below the corresponding peak, while substrate materials will show a significant rise in the background at kinetic energies below the peak, as many more electrons are inelastically scattered due to their travel through greater amounts of material. Various methods for modelling the inelastic background shape have been demonstrated [41–44], and more recently these have been tentatively extended to the analysis of nanoparticle systems [40, 45, 46]. These methods have been shown to be able to identify defects such as holes in coatings, as well as estimate average coating thickness and provide similar results to peak analysis [40, 44, 46]. As with peak analysis, background analysis may be performed using numerical modelling [42] or by the use of modelling software such as SESSA [25] or QUASES [47]. Of recent interest is the realisation that a significantly increased depth of analyses is provided by inelastic background modelling of HAXPES data [48]. Such methods may permit structural information to be determined for particles with coatings too thick for traditional XPS.

## The “ideal” case—11-nm-sized core–shell quantum dot with a CdSe core and a very thick CdS surface passivation shell stabilized with oleic acid and oleylamine ligands

Thick-shell CdSe-CdS quantum dots with a thickness of the surface passivation shell of the same size as the core exhibit exceptional optical properties which depend strongly on the particle architecture [49, 50]. However, it is very challenging to distinguish the structural homogeneity of the core and shell. To overcome this problem, the combination of HR-TEM investigations and XPS measurements aided by the simulation of the data with the software SESSA was used [51].

The size of the core/shell nanoparticles stabilized with a mixture of oleic acid and oleylamine ligands was estimated in the range between 10 and 12 nm with TEM and SAXS measurements (done with dispersed and dried QDs) that both provide solely information on the core/shell nanoobject, yet neglect the organic ligand shell. DLS, which measures the hydrodynamic diameter of the nanoobjects, provides a larger diameter of about 14.8 nm. This larger diameter usually includes the organic ligand shell which cannot be detected with the two other methods. For the XPS analysis described later, this information is crucial.

The most popular methods for determining core–shell structures are scanning transmission electron microscopy combined with energy-dispersive X-ray spectroscopy (STEM-EDX) and HR-TEM. With STEM-EDX, it was possible to verify selenium enrichment in the core, but a quantitative determination of the core and shell sizes failed. In contrast, an analysis of 245 particles with HR-TEM provides a core diameter of 3.5 nm with a standard deviation of 1.2 nm and a shell thickness of 3.8 nm with a standard deviation of 1.1 nm. The assumption of a nearly spherical CdSe-CdS quantum dot with a homogeneous shell could be confirmed.

For the analysis of the XPS data, the signal intensities of the C 1 s, Cd 3d, S 2p, and Se 3d peaks were used. Thereby, the C 1 s/Cd 3d ratio was used to determine the thickness of the organic shell, and the S 2p/Se 3d ratio for the estimation of the thickness of the CdS shell. The size of the particles cannot be reliably determined by XPS and must be input into the simulations.

For the organic ligand shell consisting of a mixture of oleic acid and oleylamine ligands, a thickness between 1.5 and 2.2 nm was estimated. This result agrees well with a rough estimation of the thickness of the organic ligand shell of about 2 nm assuming a complete surface coverage by a ligand monolayer in a stretched conformation [52, 53]. Thus, this ligand monolayer can explain the difference in the particle size obtained with TEM and/or SAXS without the ligand shell and the hydrodynamic particle diameter measured with DLS (including the ligand shell). Changes in the size of the nanoparticles between 10.5 and 11.5 nm influenced the resulting thickness of the organic ligand shell negligibly. A great source of uncertainty in this approach is the amount of adventitious carbon which is always present and differentially attenuates the intensities of the photoelectrons with different kinetic energies [54].

**Fig. 3:**
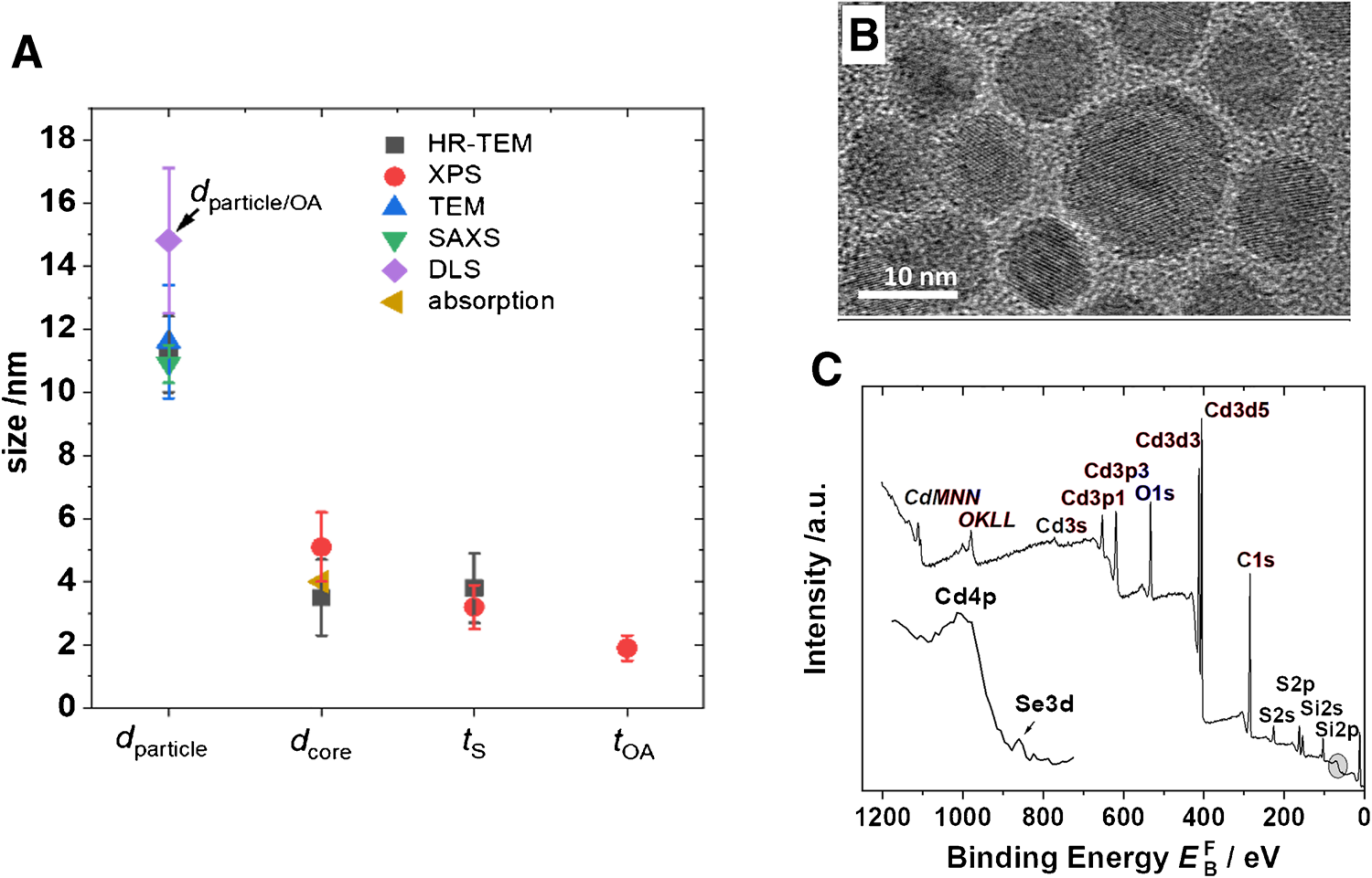
Summary of the different size results of the CdSe/CdS quantum dot with uncertainties for the diameter of the particle including the inorganic shell (*d*_particle_) which is detectable with TEM, HR-TEM, and SAXS. DLS additionally considers the ligand shell containing a mixture of oleic acid and oleylamine ligands, and herewith, the resulting particle diameter equals *d*_particle_-t_OA_. The diameter of the CdSe core (*d*_core_) and the thickness of the CdS shell (*t*_S_) are both assessable with XPS and HR-TEM while the thickness of the oleylamine ligand shell (*t*_OA_) can be obtained only with XPS (**A**). HR-TEM image of the quantum dots showing the core and the shell (**B**). XP survey spectra with the zoomed Se 3d and Cd 4p region (**C**). Data are taken from [51]

With the information on the particle size obtained with TEM and SAXS, it was possible to simulate the core–shell structure of the CdSe-CdS QDs using the intensities of the S 2p and Se 3d peaks. It must be considered that quantification of the XPS results is still challenging and shows a relatively high uncertainty [55]. For the determination of the cross section, the theoretical atom cross section was used. Recent investigations show relative differences of up to 12% in the quantification of clearly detectable components when comparing theoretical and experimental cross sections [56]. Here, the uncertainty of the spectrometer-dependent transmission function must also be considered. The other main relative uncertainty arising from the effective attenuation length of photoelectrons is also approximately 12% [57]. In summary, a relative uncertainty range of approximately 20% can be estimated for the thickness determination with XPS [58]. This is a similar uncertainty to HR-TEM. Figure 3 shows a summary of the results. Here, an inorganic shell thickness of 3.2 nm with an uncertainty of 0.7 nm was measured by XPS. From this result, a core diameter of 4.7 nm with an uncertainty of 1.1 nm was calculated. The value for the shell thickness is slightly lower to that of (3.8 ± 1.1) nm observed with HR-TEM, but it is still in the uncertainty range. Therefore, a good agreement between the results obtained with both methods can be found.

## Core–shell intermixing

Recently, it was shown that the nature of the interface between coating and core influences the properties of nanoparticles, e.g. the output colour and the quantum yield at upconverting nanocrystals [16]. Therefore, engineering such an interface is an important step for tuning the properties. Without a detailed understanding of nanointerface chemistry, the desired engineering of the interface is not possible [59]. Thereby, the structural homogeneity between the coating and the core influences the interface pattern. On the other hand, this structural homogeneity between coating and core found for many nanoparticles complicates an exact determination of the interface and the exclusion of potential intermixing with HR-TEM due to the similar structural parameter between coating and core. A good example of such a system is the CdSe-CdS quantum dot described above. With the approach using only one energy and simulations in XPS as described before, the nature of the interface cannot be resolved [51]. The simulated intensities of the relevant photoelectron peaks for particles with and without intermixing differ in the range of the uncertainty.

**Fig. 4:**
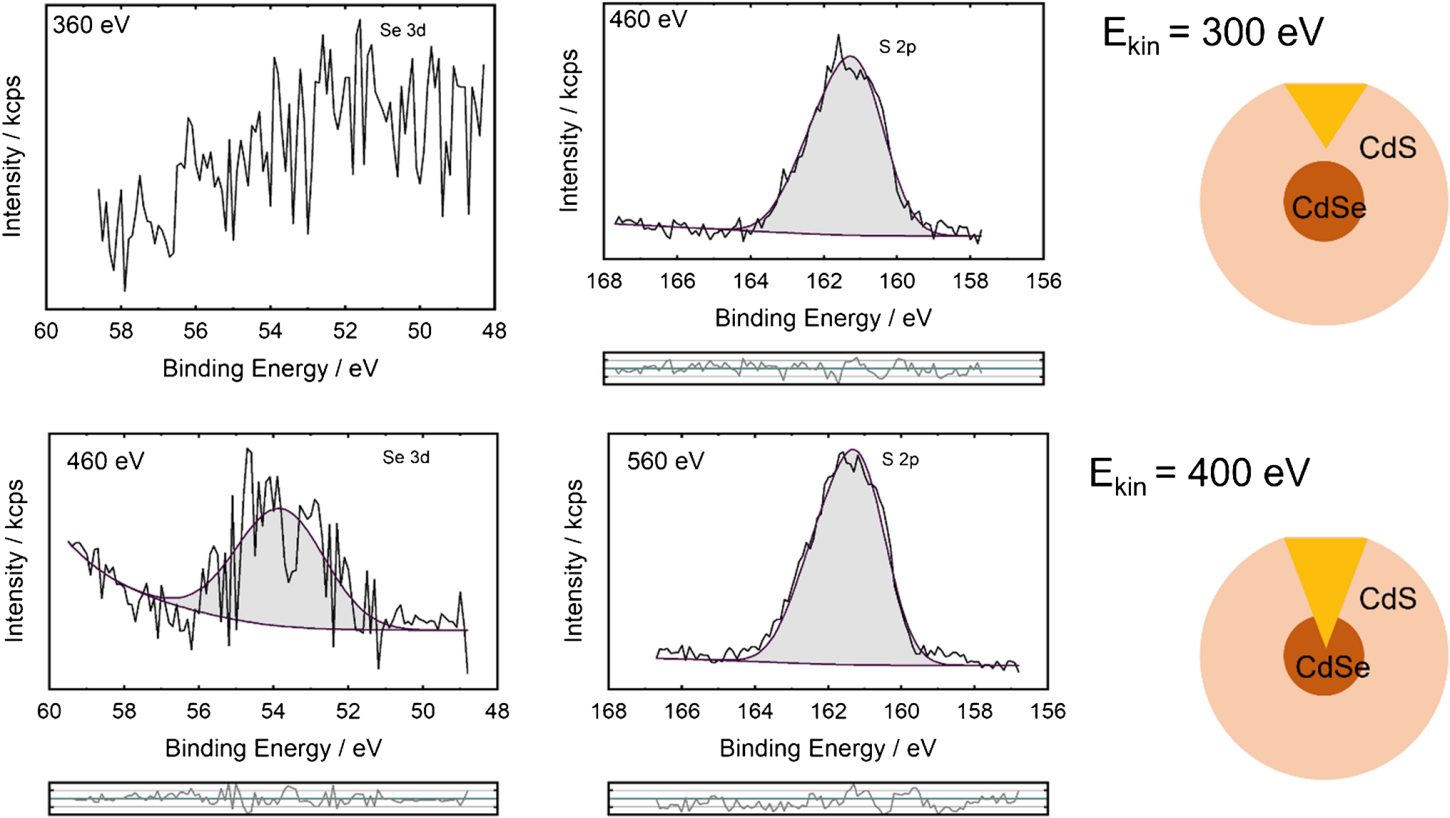
Energy resolved spectra of Se 3d and S 2p photoelectron of kinetic energies around 300 eV and 400 eV. The photon energy is given in the graphs of the spectra. The residuals between the fits and the experimental data are shown below the fitted spectra. The fit parameters are given in Table S1 (Supporting information). The different information depths correlated to the quantum dot are sketched on the right side

A possible way to investigate such intermixing is to vary the excitation energy and to look for the co-existence of the core and shell components at certain information depths. In this way, photoelectrons of these components with the same kinetic energies are measured [60]. For the CdSe-CdS nanoparticles, a small Se 3d peak was observed for kinetic energies of 400 eV and higher. For lower kinetic energies, for example 300 eV, only S 2p photoelectrons were detected, as shown in Fig. 4. A kinetic energy of 300 eV corresponds to an information depth of around 2.4 nm; the kinetic energy of 400 eV corresponds to an information depth of around 3.0 nm [61] which is in the range of the measured shell thickness, which means that at the proposed interface of the CdSe core and CdS coating, both components can be measured. This experimental approach is quite straightforward for detecting the intermixing because the occurrence of the components in the same region of the particle can be measured. On the other hand, the variation of the excitation energy is only possible at synchrotron facilities, which hinders the use of this approach for quality control or other routine measurements. This approach is suitable for verifying synthesis protocols with regard to potential intermixing.

## The ideal case—a first look on oleic acid stabilized Yb,Er-doped NaYF_4_ upconversion nanoparticles with HAXPES

For a long time, a combination of an Al Kα (hν = 1486.6 eV) with aMg Kα (hν = 1253.6 eV) has been used which allows a fast and straightforward determination of the vertical distribution of the elements and the determination of the thickness of coatings in core–shell nanoparticles [62]. The disadvantage of these experiments is that the energy difference between both excitation sources is around 200 eV and, therefore, the information depths for these sources are very similar.

To overcome this problem, instruments were developed combining X-ray excitation sources with a much larger energy difference, e.g. Al Kα (hν = 1486.6 eV) and Cr Kα (hν = 5414.8 eV) which is used in HAXPES. For particles with diameters larger than 5 nm, the composition in the near-surface region like the coating can be easily compared with the components in deeper regions of the particles like the core. Thereby, the deeper information depth which can be reached with the higher excitation energy and the consequently higher kinetic energies of the photoelectrons is used. For example, with the Cr Kα radiation, the information depth is about three times deeper compared to the Al Kα X-rays. This approach was used for NaYF_4_-based upconverting nanoparticles doped with Yb^3+^ as sensitizer ions and Er^3+^ as activator ions.

However, there is still a lack of knowledge regarding the exact structure of such type of core–shell particles in terms of intermixing of the active lanthanide ions within different shells of nanometre thickness as cation migration can occur during the shell-growing step. For example, contrary to the often used assumption that sharp interfaces are present in core-(multi)shell nanoparticles, Hudry et al. [63, 64] provided experimental evidence of partial intermixing of core and shell materials with structure-independent local energy-dispersive X-ray spectroscopy (EDXS) in a TEM and high-energy synchrotron X-ray powder diffraction for core–shell-shell (NaErYbF_4_-NaYF_4_-NaGdF_4_) and core–shell-shell-shell (NaErYbF_4_-NaYF_4_-NaGdF_4_-NaYF_4_) nanoparticles. This group also investigated cation intermixing in β-NaGdYbErF_4_@NaYF_4_ using STEM combined with EDSX and X-ray total scattering to obtain the local chemical and structural characteristics of the lanthanide ions in the core–shell upconversion nanoparticles [65].

**Fig. 5:**
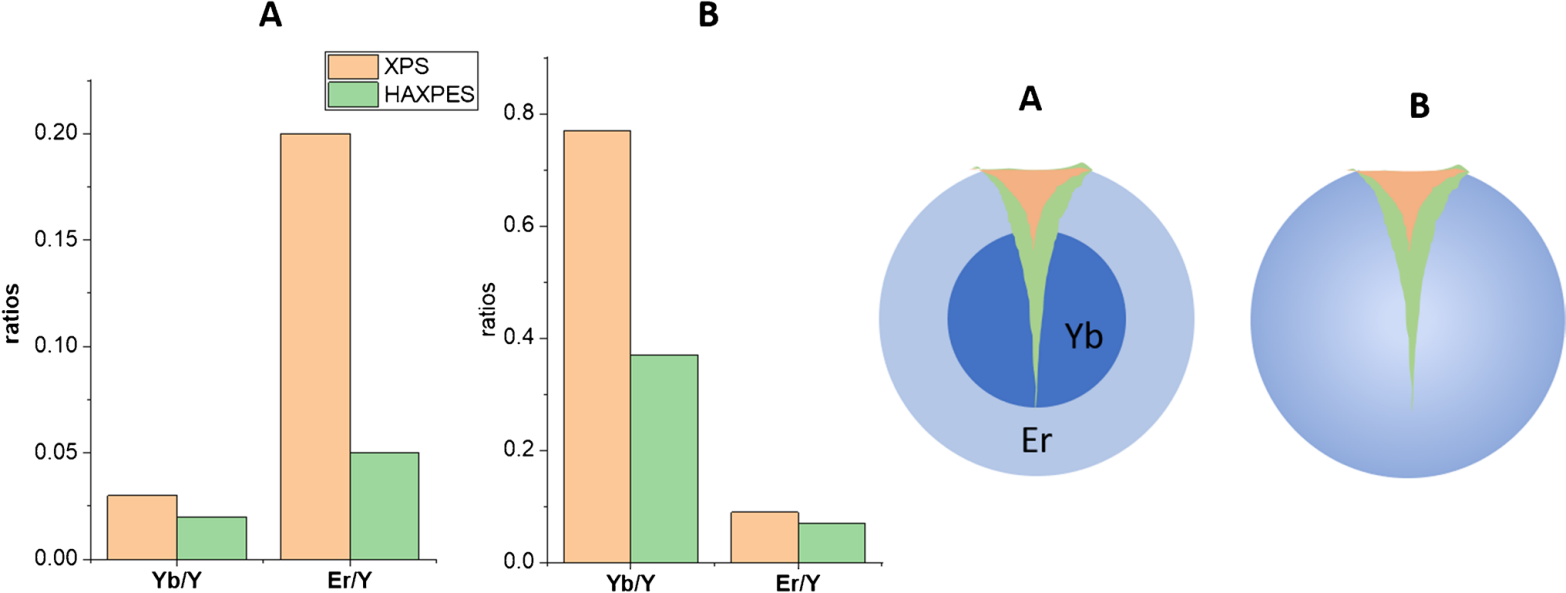
The ratios of the Yb/Y and Er/Y amounts obtained with XPS and HAXPES. In the scheme, the different information depths of XPS (orange) and HAXPES (light green) are sketched. Furthermore, the different distributions of Yb (dark blue) and Er (light blue) are indicated

Also, the combination of XPS and HAXPES, which are both available as laboratory analytical techniques and are increasingly used in the nanocommunity [66, 67], can contribute to an understanding of the average spatial distribution of the absorbing sensitizer ion and the optically active activator ion in UCNPs. This is exemplarily shown here for NaYF_4_ nanoparticles of a size of around 40 nm. One type of particle is doped with 20 mol% Yb (sample A), the other with 60 mol% (sample B). Both types contain 2 mol% Er. The survey spectra and the resulting quantification results are shown in Figure S1 and Table S2 (Supporting information), while in Fig. 5, the resulting Yb/Y and Er/Y ratios obtained with XPS and HAXPES are compared. Both Yb and Er decreased when normalized to Y with the increasing information depth for both samples, but more significant for sample A. The most significant change was observed for the Er/Y ratio between XPS and HAXPES showing that Er is in the shell of sample A. For Yb, a decrease was observed for the Yb-rich sample B, but was less pronounced. Er seems to be uniformly distributed in this sample B. Such slight changes are observed for Yb in sample A.

From these observations, a pronounced core–shell structure with an Er-rich shell is indicated for sample A, whereas in sample B, intermixing between Y, Yb, and Er with a slight enrichment of Yb in the near-surface region is consistent with the data. Such spatial distribution of the sensitizer and activator ions enables control of the donor–acceptor interaction and dynamics thereby eliminating or reducing deleterious cross-relaxation between lanthanide dopants and thus fine tuning of the optical properties [68, 69]. Additionally, the location of emitting Er^3+^ centres in the shell region enables very efficient luminescence resonance energy transfer (LRET) to organic molecules bound to the surface of the UCNPs due to the minimum distance between LRET donors and acceptors. This is beneficial for applications where UCNPs are employed as energy relay materials for surface-bound stimuli-responsive molecules [70, 71].

Summarizing, the combination of XPS and HAXPES is a promising tool to obtain a first understanding of the spatial distribution of the components in a clear manner. Further details were obtained in a recent study varying the X-ray energies in a range between 2000 and 6000 eV at the BESSY II synchrotron and using SESSA for the simulation of the spectra. For sample A at the outermost shell of the NPs, a significant enrichment of Er, a second shell with an intermixing between Yb and Er, and the Yb-doped core was found confirming the results obtained using lab-based instrumentation [72].

Another advantage of HAXPES is the access to additional transitions of higher binding energy which allows detecting photoelectrons from the same compound with a significantly different kinetic energy and, hereby, different information. For example, in survey spectra, S1s and S2p photoelectrons can be measured with an energy difference of more than 2000 eV.

## Non-centrosymmetric PTFE-PMMA core–shell nanoparticles

The investigation of the core–shell structure of polymer nanoparticles is still challenging due to the typical low contrast between core and shell caused by the similar light elements present in both regions of the nanoparticles. Thereby, methods sensitive to light elements and the chemistry of the compounds are needed for obtaining deeper insights into the core–shell structure of such kind of particles. Thus, XPS is ideally suited to answer this need.

**Fig. 6:**
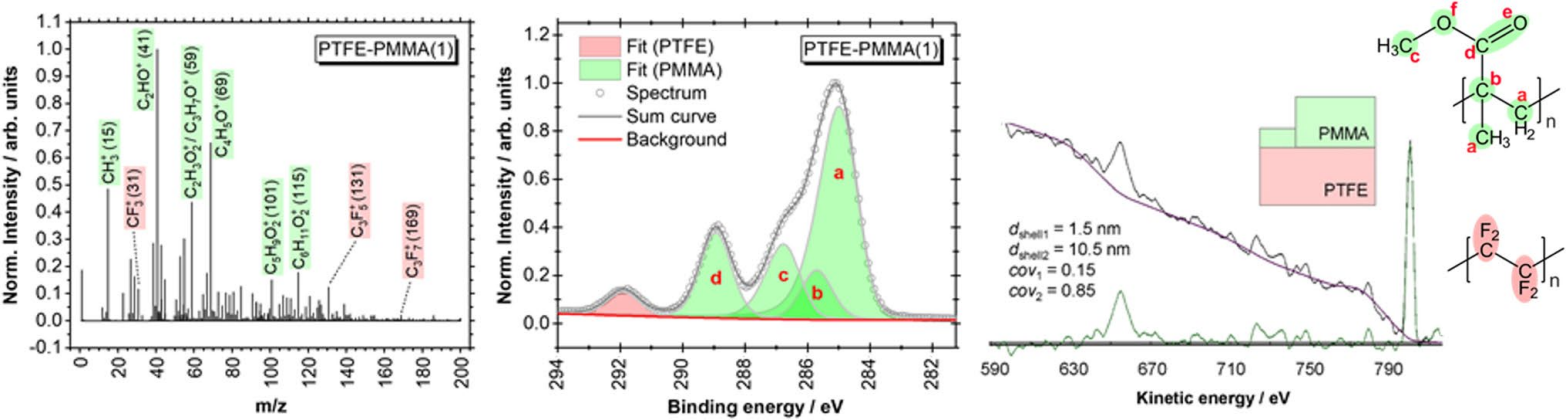
ToF–SIMS spectrum, high-resolution XPS C 1 s spectrum, and modelling of the XPS background in the F 1 s and F KLL region with QUASES of PTFE-PMMA core–shell nanoparticles with a core of 45 nm and a PMMA coating of 7.5 nm. Data are taken from [40]

Recently, thickness measurements of the coating were presented based on the intensities of the elastic peaks or on the shape of the inelastic background [40]. Particles with a PTFE core with a constant diameter of 45 nm and a coating of PMMA with a varying thickness from 7.5 to 50 nm were investigated. For the thickness determination based on the elastic peak intensities, SESSA was used. For this, the elemental peaks C 1 s, O 1 s, and F 1 s can be used. Especially, the O 1 s peak specific for the PMMA coating and the F 1 s peak specific for the PTFE core allow a clear distinction between core and coating features. Additionally, it is possible to distinguish in the high-resolution C 1 s between features originating from the PMMA coating and the PTFE core: the four C 1 s states between 285 and 290 eV are correlated with PMMA (two states of aliphatic states between 285.0 and 285.5 eV, H_3_C-O at 287 and COO at 289 eV), the peak at 292 eV is originated from CF_2_-moities of the PTFE (see Fig. 6). In the calculation of the thickness of the coating from the high-resolution C 1 s spectra, the uncertainties related to the effective attenuation length and the transmission function of the spectrometer are not relevant because of the similar kinetic energies of the photoelectrons. As an extremely surface-sensitive method with an information depth of around 1 nm, time-of-flight secondary ion mass spectrometry (ToF–SIMS) shows only very weak features correlated with the PTFE core. This observation excludes a hole in the PMMA shell.

For the estimation of the thickness of the coating with XPS, the O 1 s peak originated from the PMMA shell, the F 1 s photoelectrons from the PTFE core, and the different contributions of both compounds to the C 1 s peak (see Fig. 6) were used. Depending on the peaks which were considered in the simulation, values between 4.7 and 6.3 nm were obtained which results in a medium thickness of (5.5 ± 0.8) nm which is lower than the ones measured with the transmission mode in SEM (T-SEM) which gives a value of 7.5 nm. Thereby, the uncertainty of the thickness determination with T-SEM was rather high with nearly a factor of 2. For the microscopic investigations as the main sources of the uncertainty, the obtained pixel size, the determination of the particle boundary, the uncertainty of the used reference material, and the standard deviation of the normal size distribution of the measured particle are assumed [40]. For the presented results, the latter one contributes to the total uncertainty by far at most. This large uncertainty could indicate an acentric position of the cores. For the nanoparticles with a coating of ca. 20 nm, the uncertainty of the thickness determination with electron microscopy is ca. 33%. For these particles, coatings with a thickness of (11.0 ± 1.6) nm were determined with XPS. These values are all below the thicknesses determined with SEM even if the uncertainties are considered. Such a discrepancy between the electron microscopy and the XPS results indicates inhomogeneities in the coating.

More insight into the potential inhomogeneities of the coating was gained with the analysis of the inelastic background with QUASES. In contrast to the former simulation which regards the elastic photoelectron peaks, hereby the fraction of photoelectrons is scattered inelastically while passing through the sample towards the detector. This scattering leads to tails on the high binding energy side of the elastic peaks and forms the inelastic background. The shape of this inelastic background is strongly influenced by the nanostructure of the sample [44, 45, 73]. To analyse the inelastic backgrounds, the software package QUASES was used. The advantage of this approach is the higher information depth by a factor of 2 to 3 [74] and the possibility to distinguish between non-centrosymmetric structures from an ideal structure. Herewith, a simulation with a minimum coating thickness of 1.5 nm and a maximum coating thickness of 10.5 nm gave the best results for the smallest nanoparticles (T-SEM 7.5 nm), and, for the thicker coating (T-SEM 20 nm), thicknesses between 4.0 and 13.0 nm were found when using the regions on the high-energy binding side of the F 1 s photoelectron and *F KLL* Auger peaks. It must be noted that the best results for the QUASES analysis were obtained using two different thicknesses for the coating whereas the SESSA simulation provides only one thickness. As expected, the results obtained with SESSA are within the range of the minimum and maximum thickness received by the QUASES analysis. Whereas the results of the smallest nanoparticles are consistent between the determination of coating thickness with T-SEM and XPS with QUASES, this is not the case for the larger particles. Here, XPS underestimated the thickness of the coating with both methods.

**Fig. 7:**
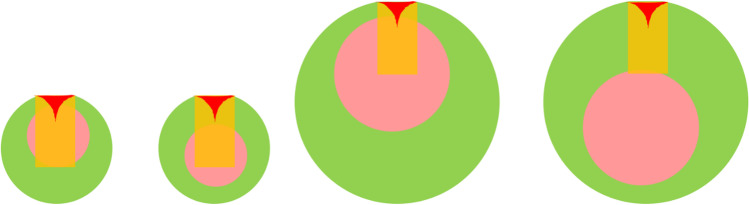
PTFE-PMMA nanoparticles of different sizes orientated differently to the detector. The different information depths of SESSA (peak intensities: shown in red) and QUASES (background simulation: shown in orange) are sketched

This observation can be explained by the orientation distribution of the non-centrosymmetric nanoparticles relative to the detectors (see Fig. 7) [31]. In a randomly oriented population, a fraction of the nanoparticles will be positioned so that the core is detected with XPS; for other particles, the core is below the information depth of both methods and cannot be observed in the experiments. This effect of inhomogeneity in coating thickness always leads to an underestimation of the thickness of the coating using XPS and an assumption of uniform coating. HAXPES which gives a higher information depth than XPS is a good possibility to identify this effect and to measure larger particles. HAXPES data often has a larger region of inelastic background not influenced by photoelectron or Auger peaks than XPS data. This additional range enables a more precise simulation of depth distributions. Such regions can be more easily found in the HAXPES spectra particularly for core levels with a binding energy higher than 1000 eV. It must be noted that simulation of the photoelectron peak intensities and of the inelastic backgrounds requires different preparation protocols of the nanoparticles. For the simulation of the photoelectron peak intensities, the influence of the substrate should be minimized. Layers of nanoparticles thicker than one monolayer can be considered in the simulation. In contrast, the simulation of the inelastic background is easiest for a single-particle layer. Therefore, the influence of the substrate must be taken into account. Only the inelastic background of photoelectrons which are not influenced by structure in the photoelectron spectra of the substrate can be used. Hence, only the background on the high binding energy side of the F peaks was simulated in the examples given here because the background of the C and O peaks can be influenced by the typical contamination of the substrate. Further information about different preparation methods for polymer nanoparticles like drop casting, spin coating, or cryofixation was published recently [75].

**Fig. 8:**
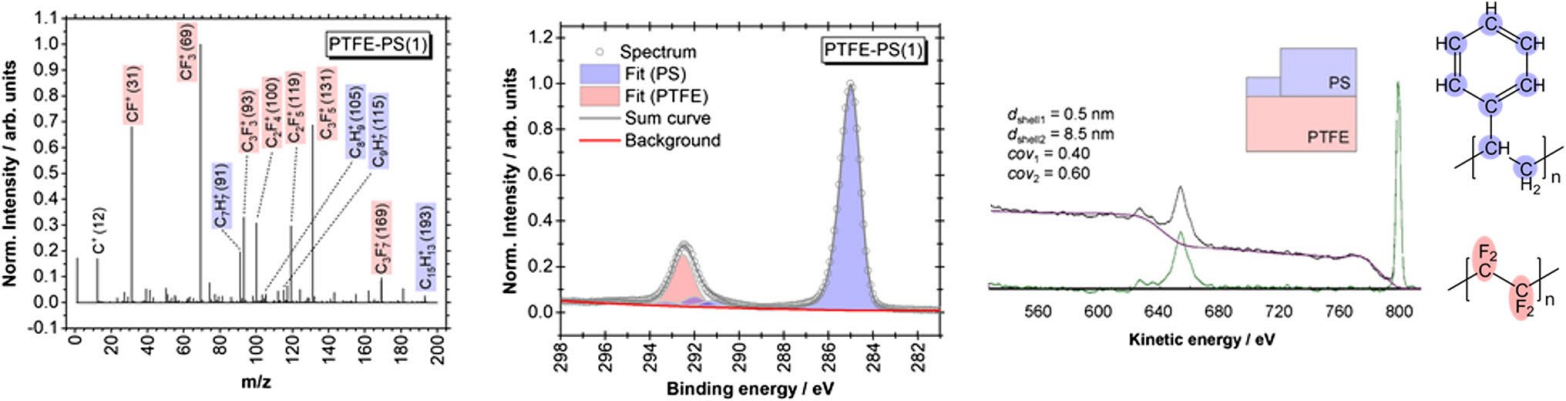
ToF–SIMS spectrum, high-resolution C 1 s spectrum, and modelling of the background in the F 1 s and F KLL region with QUASES at PTFE-PMMA core–shell nanoparticles with a core of 45 nm and a PS coating of 4 nm. Data are taken from [40]

Another example of such polymer nanoparticles are PTFE cores coated with PS. As for PTFE-PMMA, cores with a diameter of 45 nm were used. The thickness of the PS coating varied from 4 to 51 nm. Here, the C 1 s peak at 284.5 eV, typical of aromatic carbon, and the peak at 292 eV are the peaks for carbon bonded to fluorine in PTFE, and the F 1 s peak as described above can be used for the calculation of the thickness of the coating. A small amount of uncertainty arose from interference from the π to π* shake-up satellite of PS at 291 eV which is typical for aromatic carbon (Fig. 8). Surprisingly, for all samples, the features of the PTFE core can be detected, even for the sample with a nominal coating thickness of 51 nm. This observation can only be explained with an inhomogeneous or incomplete coating. To verify this assumption, time-of-ToF–SIMS measurements were performed. As expected from the XPS results, for all samples, PTFE features were observed clearly. In contrast, for the PTFE-PMMA samples, no peaks (or only weak peaks in the case of the thinnest PMMA shell) correlated with PTFE were visible. QUASES simulation of the PTFE-PS particles confirmed that a significant part of the shell is thinner than 1 nm.

From these observations, it can be expected that the interaction of the polymer nanoparticles with their environment is dominated by the PMMA coating for the PTFE-PMMA core–shell particles, whereas for the PTFE-PS particles the PTFE core must be considered due to the incomplete coating. These results underline the importance of such surface analytical investigations of the chemical composition for the understanding of the properties of nanoparticles.

Another exciting possibility to obtain insights into the internal structure of polymer nanoparticles provides modern Ar cluster sputtering which was used for the same PTFE-PMMA core–shell particles presented here. It was definitively shown that the nanoparticles have a complete PMMA shell but with randomly positioned internal cores, which was an assumption in the QUASES simulation [76]. Using the argon cluster sputtering approach, the total volume fraction of PTFE was accurately measured and, with knowledge of the sputtering yield, the particle diameter could also be estimated. For organic nanoparticles and sub-micron particles such as drug delivery systems, this combination of XPS and argon cluster sputtering is extremely promising.

## Sources of uncertainty

In this part, we want to discuss the sources of uncertainty for investigating core–shell structures with XPS. The data reduction for quantitative results is model based in XPS and, as for all model-based approaches, the used model must be validated. Thus, all uncertainties of the used model must be considered in the interpretation of the quantitative results. Usually, spherical particles with sharp boundaries between the different regions of the nanoparticles are assumed. In reality, this is not always the case. Whereas the morphology of a particle can be relatively easily found with microscopical methods, the sharpness of the boundaries is often unknown. Investigations about intermixing are still challenging, and XPS with variable excitation sources can help to overcome this gap.

Another important source of uncertainty is the effective attenuation length. This parameter describes the surface sensitivity of the methods which is crucial for the thickness determination of the coating. Usually, necessary algorithms for the calculation are implemented in the simulation tools like SESSA [25] or in separate software packages [61]. An experimental determination of effective attenuation lengths is complicated and too time-consuming for most analytical work. The relative uncertainty is estimated in the range of 10 to 15% [57, 77].

Although XPS is a quantitative method, quantification is not easy and straightforward [55]. The transmission function of the spectrometer must be known, and several approaches for their determination are published [78–80]. Furthermore, the sensitivity factors used are another source of uncertainty. Empirical and theoretical sensitivity factors can be used. Here, the relative uncertainty can be estimated in the same range as the effective attenuation length in the range of 10 to 15%. Finally, the peak area determination relies upon the correct choice of background and this contributes to a relative uncertainty of at least 10%, dependent upon the details of the sample, the peak intensity, and the care taken during data analysis.

**Fig. 9:**
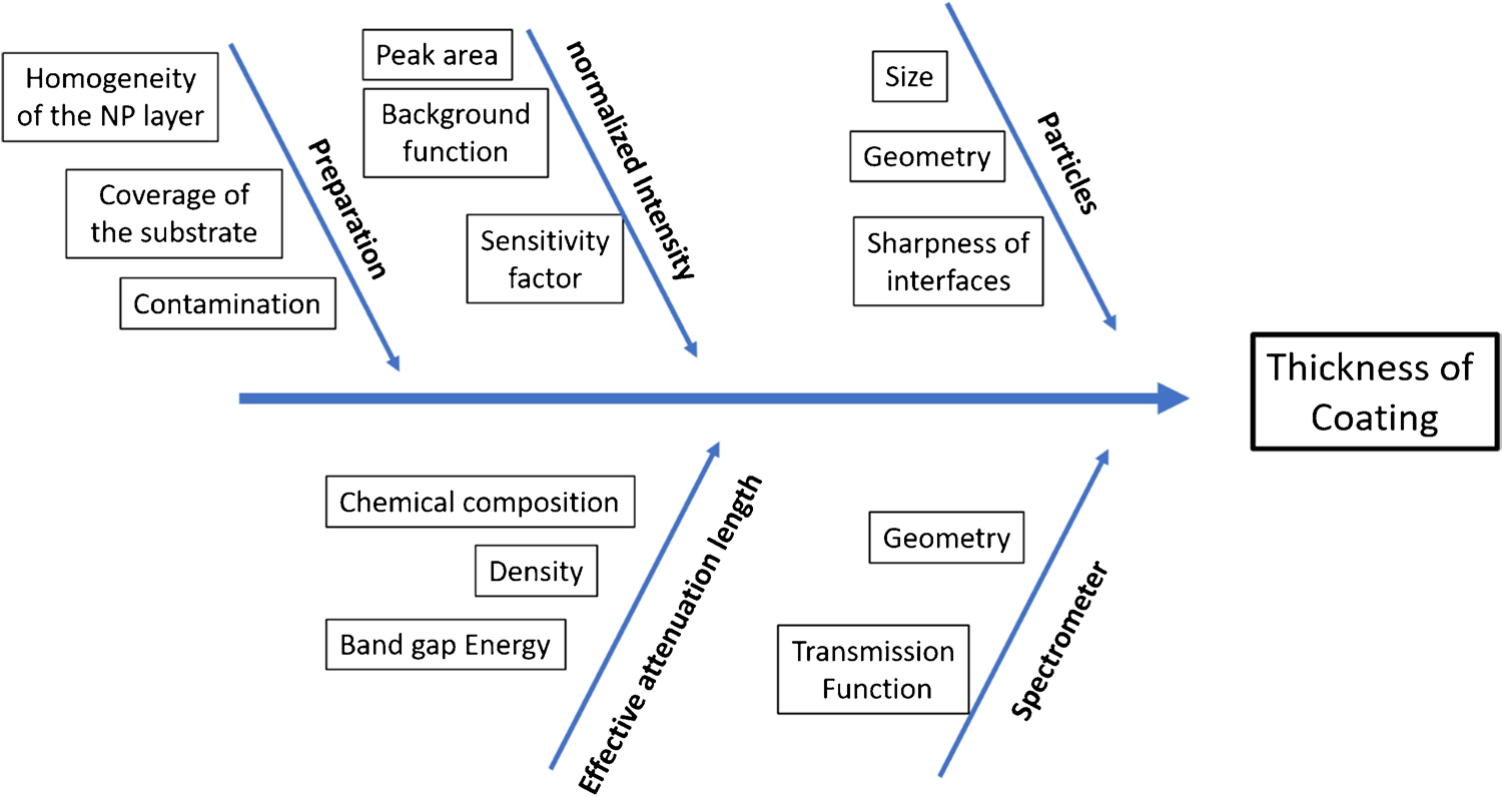
Cause-effect diagram for the determination of the coating thickness with peak intensities

Another critical factor for the success of the experiments is sample preparation. Without a suitable sample preparation method, reliable results cannot be obtained. Appropriate methods were described recently [75, 81]. In Fig. 9, a cause-effect diagram is shown, which summarizes all these sources of uncertainties. A deep understanding of all these factors with a quantitative uncertainty budget is necessary for a reliable determination of the thickness of the coating. On the first view, the whole relative uncertainty of about 30% seems to be rather high, but a critical statistical evaluation of TEM results provides uncertainties in the same range [51]. In spite of this relatively low accuracy, the precision of XPS is excellent: interlaboratory exercises demonstrate that using standard procedures, the variability in repeat analyses of the same sample is close to 1% [82], even for instruments in different laboratories. Therefore, the ability to detect differences in the thickness of 0.1 nm or less between different samples is feasible. This is evidenced by the unparalleled ability of XPS to measure the thickness of silicon oxide layers on silicon [83].

## Conclusions

The examples presented in this paper show the possibilities of XPS to gain knowledge about nanoparticle coatings. With its sensitivity to light elements, for the valence states of nearly all elements (except H and He) and the understanding of the correlation between peak intensity, quantitative composition, and morphology of the sample, XPS is an ideal addition for the microscopical investigations which are typical for nanoscale analytics. In contrast to transmission or scanning electron microscopy with a lateral resolution in the sub- or nanometre range, XPS is an ensemble technique with a lateral resolution of several micrometres. In contrast, it is an inherent nanoscale technique in the vertical axis. Hence, it is a tool for nanoanalytics and complementary to electron microscopy.

The strength of XPS compared to other methods for the investigation of core–shell particles is the chemical sensitivity. The different valence states and, herewith, the different carbon compounds like PTFE, PMMA, and PS can be characterized. Furthermore, information about organic ligand shells can be obtained which are not or only minimally accessible with other methods like (HR-)TEM, SAXS, or EDS. Other methods like mass spectrometry usually need the removal of the coating prior to its analysis. These organic ligand shells can determine the properties of the particles, like hydrophobicity or solubility [56]. Therefore, the need for methods investigating the surface chemistry of nanoparticles, e.g. for risk assessment, is obvious [84–86]. Here, XPS with its capabilities for investigating coatings of nanoparticles can make an important contribution.

XPS with variable X-ray sources can be used to obtain more information about the intermixing between the components of the core and the coating. Usually, such instruments are available at synchrotron radiation sources, but equipment combining usually Al Kα (*E* = 1486.6 eV) and X-ray sources with higher energy, so-called HAXPES sources, are becoming more popular in laboratories. Thereby, measurements varying the information depth of the photoelectrons are possible which broadens the application of XPS for core–shell nanoparticles. A fast overview of the components in the coating and in the core can be obtained. Further information about intermixing can be expected using the simulation software for spectra. Larger regions without any peaks from photo- or Auger electrons allow a more detailed and reliable simulation of the background. An alternative to this non-destructive approach is soft sputtering with modern Ar cluster ion guns. Therefore, an understanding of the sputtering process is necessary [76]. All in all, these recent technological innovations can boost the application of XPS for core–shell nanoparticles, especially addressing the question of intermixing which is often controversially discussed [16].

Ideally, a combination of complementary methods investigating the size, morphology, architecture, and chemistry of core–shell nanoparticles is necessary to obtain a comprehensive insight into their nature. Furthermore, single-particle methods like electron microscopy coupled with spectroscopic methods like EDS should be combined with ensemble methods like SAXS or XPS. It must be noted that single-particle methods give a representative image about the nanoparticles only with a rigorous statistical evaluation. On the other hand, ensemble methods often need the input of microscopic methods about the size or the morphology of the particles. Summarizing, XPS can be an important piece in the puzzle of understanding the properties of core–shell nanoparticles and help us to obtain a further knowledge especially about their chemistry.

## Supplementary Information

Below is the link to the electronic supplementary material.

Supplementary file1 (DOCX 387 KB)
