# Supplementary material for: Composition, thickness, and homogeneity of the coating of core–shell nanoparticles—possibilities, limits, and challenges of X-ray photoelectron spectroscopy
Source: Anal Bioanal Chem. 2022 Apr 26;414(15):4331–45. doi: 10.1007/s00216-022-04057-9 (PMC9142455; doi:10.1007/s00216-022-04057-9)
Supplement: Supplementary file 1 — Supplementary file1 (DOCX 387 KB) [file 216_2022_4057_MOESM1_ESM.docx]

# Supporting Information

# Composition, thickness, and homogeneity of the coating of core-shell nanoparticles – possibilities, limits, and challenges of X-ray photoelectron spectroscopy

J. Radnik^1^, X. Knigge^1^, E. Andresen^2^, U. Resch-Genger^2^, D. J. H. Cant^3^, A. G. Shard^3^, and C. A. Clifford^3^

^1^ Bundesanstalt für Materialforschung und -prüfung (BAM), Division 6.1 „Surface analysis and interfacial chemistry, Unter den Eichen 44-46, 12203 Berlin, Germany

^2^ Bundesanstalt für Materialforschung und -prüfung (BAM), Division 1.2 „Biophotonics“, Richard-Willstätter-Str. 11, 12489 Berlin, Germany

^3^National Physical Laboratory, Surface Technology Group, Teddington, TW11 0LW, UK

Details for the measurement conditions, data reduction and data interpretation of…

…the “ideal” case – 11 nm-sized core/shell quantum dot with a CdSe core and a very thick CdS surface passivation shell can be obtained in:

Weigert, F., Müller, A., Häusler, I., Geißler, D., Skrobin, D., Krumrey, M., Unger, W.E.S., Radnik, J. & Resch-Genger, U. Combining HR-TEM and XPS to Elucidate the Core-Shell Structure of ultrabright CdSe/CdS Semiconductor Quantum Dots, *Scientific Reports* **10,** 20712, doi:10.1038/s41598-020-77530-z (2020)

## …the polymer particles PTFE-PMMA and PTFE-PS in:

Müller, A., Heinrich, T., Tougaard, S., Werner, W.S.M., Hronek, M., Kunz, V., Radnik, J. et al. Determining the Thickness of the Shell of Polymer Core-Shell Nanoparticles by X-ray Photoelectron Spectroscopy, Secondary Ion Mass Spectrometry, and Transmission Scanning Electron Microscopy. *J. Phys. Chem. C* **123**, 29765-29775 (2019).

*Experimental details of the energy-resolved measurements at the CdSe-CdS core shell nanoparticles*

Energy resolved XPS measurements were carried out at the synchrotron BESSY II at HZB. The spectra were acquired at the HE-SGM monochromator dipole magnet CRG beamline at photon energies given in Figure 4. The spectra were measured in normal emission, the angle between the X-ray source and the sample normal was 45°. For the detection of the spectra a Scienta R3000 analyzer was used. The pass energy was 50 eV for the measurements. For the background a modified Tougaard background was used. The peaks were fitted with the sum of Gaussian-Lorentzian curves. The binding energy was referenced to the C 1s peak at 285 eV.

The effective attenuation lengths of the photoelectrons were determined with the NIST standard reference database (version 1.3, SRD 82).

The samples were prepared by dropping a suspension of the nanoparticles in hexane solution on a cleaned Si wafer.

Table S1:

The fitting parameter for

S2p with 460 eV excitation energy:


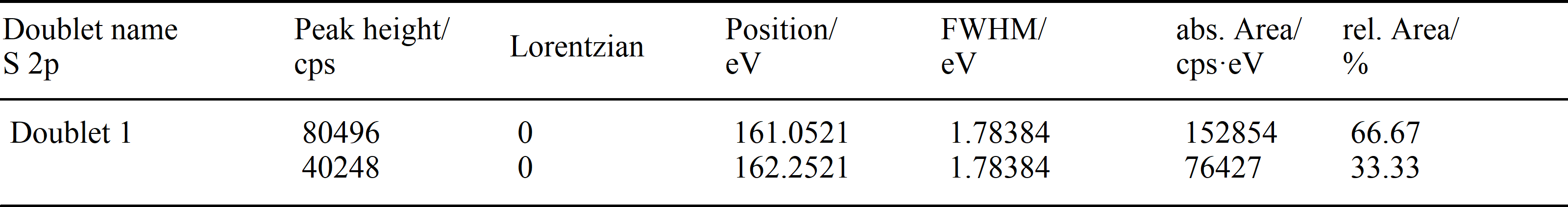


S 2p with 560 eV excitation energy


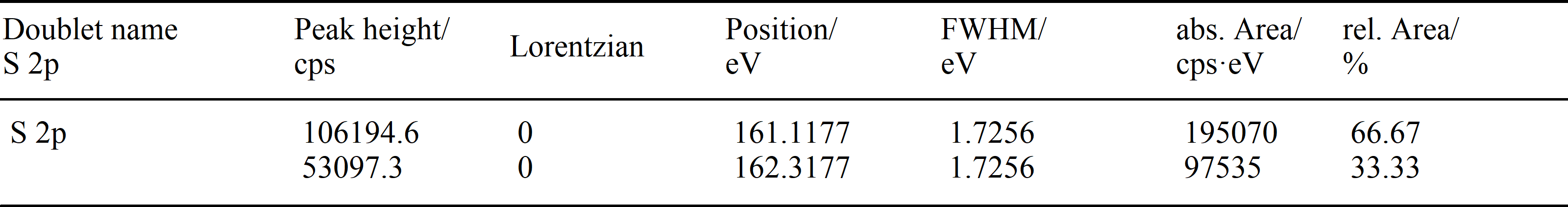


Se 3d with 460 eV excitation energy


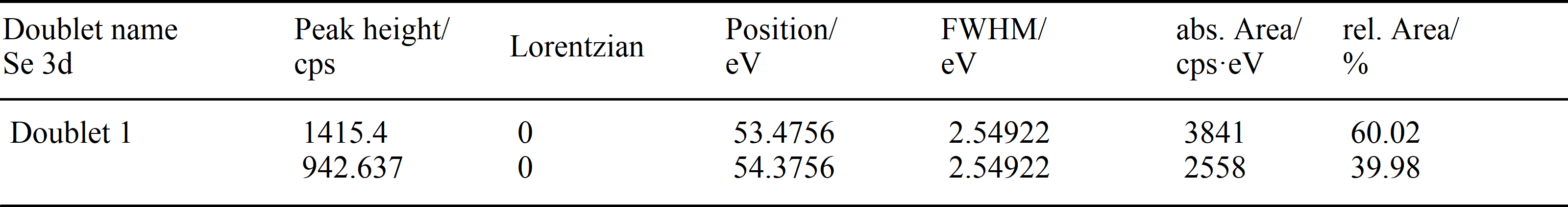


*Experimental details of the XPS andHAXPES measurements at the upconversion core shell nanoparticles*

These measurements were carried out at a ULVAC-PHI Quantes with an Al Kα-radiation source (1486.6 eV) and a Cr Kα-radiation source (5414.8 eV). The spectra were measured at an emission angle of 45°, the angle between the source and the sample was 49°. For the detection of the spectra a spherical analyzer with a multichannel resistive plate was used. The data processing was performed with MultiPak (version 9.9.1.1). For the background a Shirley background was used. For the quantification the empirical relative sensitivity factors provided by PHI were used.

The samples were prepared by dropping a suspension of the nanoparticles in hexane solution on a Si wafer.


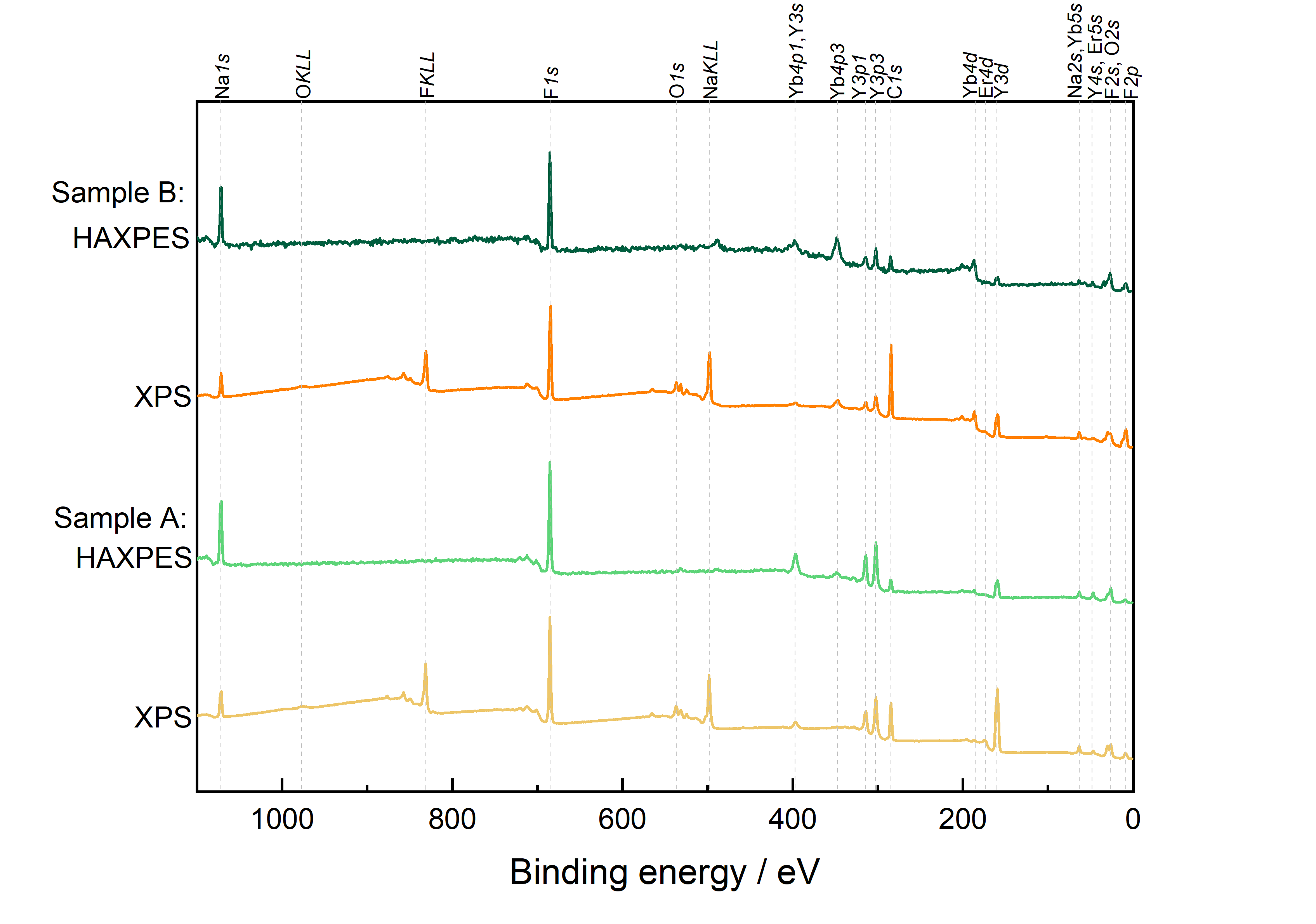


Figure S1: Survey spectra (XPS and HAXPES) of both samples.

*Table S2: Quantification results obtained with MultiPak 9.9.1.1.*

| **Peaks** | **Sample A** | | **Sample B** | |
| --- | --- | --- | --- | --- |
|  | **XPS** | **HAXPES** | **XPS** | **HAXPES** |
| **Na 1s** | 8.2 | 12.5 | 6.0 | 12.1 |
| **Y 3d** | 11.3 | 15.0 | 3.5 | 7.1 |
| **F 1s** | 34.8 | 42.8 | 26.3 | 39.8 |
| **Yb 4d** | 0.3 | 0.3 | 2.7 | 2.6 |
| **Er 4d** | 2.1 | 0.4 | 0.7 | 0.4 |
| **C 1s** | 39.0 | 24.4 | 57.8 | 31.7 |
| **O 1s** | 4.3 | 4.6 | 3.0 | 6.3 |
